# Raw Spectral Filter Array Imaging for Scene Recognition

**DOI:** 10.3390/s24061961

**Published:** 2024-03-19

**Authors:** Hassan Askary, Jon Yngve Hardeberg, Jean-Baptiste Thomas

**Affiliations:** 1 Department of Computer Science, NTNU—Norwegian University of Science and Technology, 2815 Gjøvik, Norway; hassan.askary@outlook.com (H.A.); jean@spektralion.com (J.-B.T.); 2 Spektralion AS, 2815 Gjøvik, Norway; 3 Imagerie et Vision Artificielle (ImVIA) Laboratory, Department Informatique, Electronique, Mécanique (IEM), Université de Bourgogne, 21000 Dijon, France

**Keywords:** spectral filter array, scene recognition, convolutional neural networks

## Abstract

Scene recognition is the task of identifying the environment shown in an image. Spectral filter array cameras allow for fast capture of multispectral images. Scene recognition in multispectral images is usually performed after demosaicing the raw image. Along with adding latency, this makes the classification algorithm limited by the artifacts produced by the demosaicing process. This work explores scene recognition performed on raw spectral filter array images using convolutional neural networks. For this purpose, a new raw image dataset is collected for scene recognition with a spectral filter array camera. The classification is performed using a model constructed based on the pretrained Places-CNN. This model utilizes all nine channels of spectral information in the images. A label mapping scheme is also applied to classify the new dataset. Experiments are conducted with different pre-processing steps applied on the raw images and the results are compared. Higher-resolution images are found to perform better even if they contain mosaic patterns.

## 1. Introduction

Scene recognition is a challenging computer vision task that entails classifying an image into various scene categories based on the present visual information [1]. In contrast to object recognition, it requires modeling of the entire context in the image, including object presence, spatial location, illumination condition, viewing angle, distance, and scale [1,2]. It has applications in autonomous driving, robotics [3,4], video surveillance [5,6,7], augmented reality [8], and image retrieval [9,10]. It is a difficult task for the machine due to the large interclass similarities and intraclass variations present in different scene categories such as *book store*, *library*, and *archive*, all having similar objects present in the image and having similar layouts and ambient conditions [2].

In this work, the problem of scene recognition in raw spectral filter array (SFA) images is investigated using convolutional neural networks (CNN). The goal is to assess the effectiveness of using raw SFA images for this task. Usual spectral imaging acquisition setups consist of either capturing images in different spectral bands by cycling through multiple optical filters or by capturing the whole multispectral range using diffraction gratings, but one line at a time. Both of these approaches have a limitation of high acquisition time depending on the number of spectral bands or image size. They are also prone to artifacts due to movement during acquisition. Spectral filter array (SFA) technology [11] solves both of these problems by capturing the multispectral image in a single exposure at the expense of spatial resolution. It is similar to the color filter array (CFA) in RGB cameras. It is based on a single sensor overlayed with a Bayer-like pattern of different spectral filters with different spectral sensitivities over each pixel. The number of spectral bands used depends on the design of the SFA pattern. Demosaicing must be performed to reconstruct the full-resolution multispectral image, lowering the spatial resolution compared to other spectral imaging methods. Demosaicing is an ill-posed problem, where interpolation is required to reconstruct the missing intensity values for each pixel. It also introduces estimated values that might be incorrect, which requires extra processing to rectify. This rectification process is scene-specific and requires identification of the targeted scene beforehand. To avoid these problems, in this work, scene recognition is performed on SFA images without demosaicing them. This speeds up the acquisition time even further because no pre-processing step is applied, and this also enables exploitation of spectral bands for scene classification. Furthermore, a large and diverse raw SFA dataset for scene recognition is introduced, and finally CNN models are investigated to perform scene recognition in raw SFA images.

One of the earliest works in scene recognition is by Szummer and Picard [12]. They classified scene images into *indoor* or *outdoor* categories based on low-level image features. They used the Ohta color space and multi-resolution simultaneous autoregressive model [13] to represent color and texture information. They computed these features on sub-blocks of the input image and then classified them; finally, they combined the classification result from each sub-block to obtain a final prediction using the K-nearest neighbor model. The approach was tested on a fairly small dataset of 1300 images and only for binary classification. Oliva and Torralba [14] proposed the Spatial Envelope representation for general scene classification. It is a global feature representation of the scene image. It describes a scene using five perceptual properties: naturalness, openness, roughness, ruggedness, and expansion. The classification prediction is performed using K-nearest neighbors. The authors also assembled a large dataset consisting of 8100 images over 4000 categories of natural scenes and 3500 categories of urban scenes. The Spatial Envelope representation does not consider local object information, making it sensitive to occlusions and spatial variations [1]. To overcome this, the Bag-of-Visual-Words (BoVW) framework was introduced in which local feature descriptors are extracted from the image. Then, the feature descriptors are quantified in terms of visual words. The image can now be classified on the basis of the frequency of occurrence of these visual words. Fei-Fei and Perona [15] proposed an approach where the scene image is first represented as a bag of codewords, then a probabilistic Bayesian hierarchical model is learned for each class. The model can learn to categorize the local regions of the image in an unsupervised way. It requires only the ground truth categories of the images for training. The model showed limitations in classifying complex indoor scenes because the BoVW approach does not take into account the spatial relationship of local features. To improve on this, Lazebnik et al. [16] proposed Spatial Pyramids. They repeatedly subdivided the image and computed the histogram of the local image features over the subregions. This hierarchical multiscale representation is a generalized form of the BoVW framework capturing spatial information. However, it is not invariant to geometric variation.

The recognition of outdoor scenes is easier than the recognition of indoor scenes. Indoor scene recognition is more difficult because of high inter-class variability present in the images, such as images of library, archive, and book store look similar. Quattoni and Torralba [17] tackled improving performance in indoor scene classification tasks. They devised a prototype-based model that combines global and local discriminative features. The model is based on the idea that images containing similar objects must have similar labels and that the presence of some objects in a scene is more important than that of others for determining the scene label. The authors created prototype images by annotating discriminative regions of interest in those images. Then, spatial pyramids were used extract features from query image, and the features were compared with the prototype regions of interest for similarity.

Until recently, approaches to recognizing scenes have relied on handcrafted features and classical machine learning models such as support vector machines [18] and K-nearest neighbors. Krizhevsky et al. [19] demonstrated the feasibility and superior performance of using deep convolutional neural networks (CNNs) in the large-scale image classification task, ushering in a new era in computer vision. It allows for end-to-end learning of the classification task. The CNN model is composed of a set of convolution layers and then another set of fully connected layers. The convolution layers extract features from the dataset, while the fully connected layers perform the classification. The entire network is tasked with minimizing the loss function using gradient descent, enabling it to automatically learn to extract useful discriminative features and perform classification. Deep learning models outperform classical methods by a large margin; however, they require large datasets and more time for training.

Zhou et al. [20] used the CNN model for scene recognition and also introduced a new large-scale scene recognition dataset called Places [21] with 10 million images. The Places-CNN model achieved state-of-the-art performance on existing benchmark datasets and on the new Places dataset. After this, many variants of deep learning models have been used for scene recognition tasks, improving performance, and pushing the state of the art forward. Some notable works include DAG-CNN [22], which uses a hierarchical CNN model to improve the extraction of local feature and gradient flow, and GAP-CNN [23], which replaces fully connected layers with global average pooling layers, biasing the model to attend to class-specific regions of the scene and reduce the number of learnable parameters.

Most of the work in scene recognition uses RGB images. The performance of scene recognition algorithms can be improved by exploiting additional spectral bands. Brown and Süsstrunk [24] proposed an extension of the Scale-Invariant Feature Transform [25] descriptor for multispectral images for scene recognition. Xiao et al. [26] extended the CENTRIST [27] descriptor to use multispectral images for scene recognition by capturing joint channel information from the RGB and NIR channels. Recently, Sevo and Avramović [28] used the convolutional neural network (CNN) on multispectral images of scenes to predict the scene label. However, in all of these works, one point to note is that the dataset consists of images with only four channels, RGB+NIR. Additionally, Elezabi et al. [29] collected a dataset of raw SFA images of textures to perform texture classification using CNNs and also investigated the impact of different illumination and exposure variations on performance.

To the best of our knowledge, there is no dataset of raw spectral filter array images of indoor and outdoor scenes. Also, to the best of our knowledge, there has been no prior work solving task of scene recognition in raw SFA images using CNNs.

This paper is organized as follows. Section 2 covers the details of the novel raw SFA dataset. Section 3 introduces our architecture to solve scene recognition in raw SFA images based on CNNs. The results are presented in Section 4, and finally the conclusions are presented in Section 5.

## 2. Dataset

**Figure 1:**
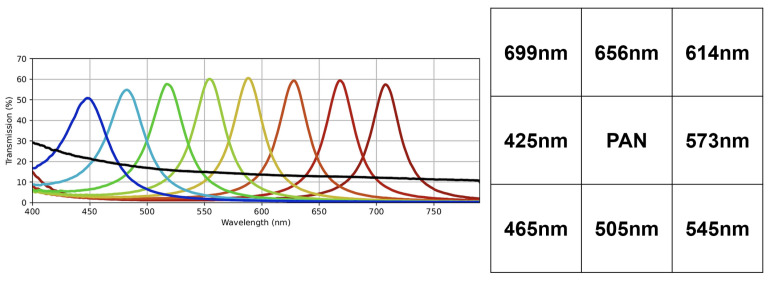
Arrangement of spectral bands in SFA pattern of SILIOS CMS-C sensor as well as transmission and wavelengths of spectral bands of each filter. Reproduced from [29,30,31].

**Figure 2:**
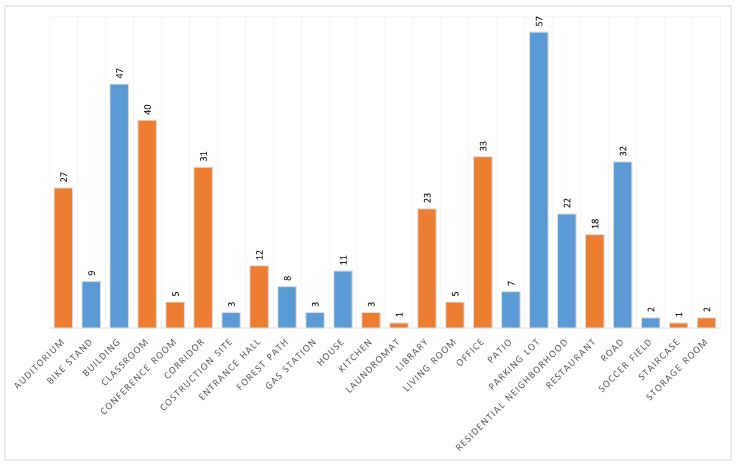
All scene categories and their sizes in our raw SFA scene recognition dataset. Blue color means outdoor scene and orange color means indoor scene.

**Figure 3:**
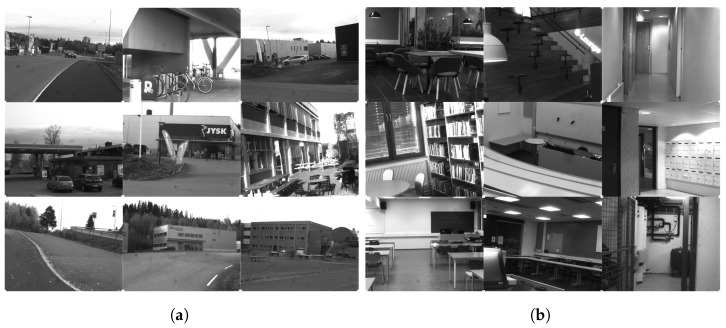
Random sample of indoor and outdoor datasets. (**a**) Outdoor raw SFA images. (**b**) Indoor raw SFA images.

A novel dataset consisting of raw SFA images of indoor and outdoor scenes was collected, entitled CID:Places. The dataset was collected using the SILIOS CMS-C SFA camera [30]. It captures nine bands ranging from 430 nm to 700 nm with a resolution of 1280 × 1024. Figure 1 shows the arrangement of the SFA pattern along with the spectral bands of the sensor. The dataset is comprised of various indoor and outdoor scenes. All images are 8-bit raw and mosaiced. Each image has a label indicating whether it is an indoor or an outdoor scene, as well as the specific scene category. In total, it has 402 raw SFA images, of which 201 are indoor scenes and the other 201 are outdoor scenes. It consists of 24 specific scene categories that are shown together with the number of images in Figure 2. Figure 3a shows a random sample of outdoor images, and Figure 3b shows a random sample of indoor images.

The SILIOS CMS-C camera was mounted on a Joby GorillaPod 5K tripod. To capture the scenes, a 12.5 mm lens with a widest aperture of f/1.3 was used. The camera was connected to a Windows laptop with the IDS uEye Cockpit [32] program running. Two people were needed to carry out the captures. One person framed the picture, monitored the histogram, modified the parameters, triggered the capture on the laptop, and the other person held the camera setup. All images were captured in 8-bit sensor raw using the uEye Cockpit 2023 software. It allows for live view of what the camera is seeing along with the image histogram. It also performs live auto-exposure to properly expose the images, although in some extreme lighting situations the lens aperture and focus were manually adjusted.

In the dataset, the *classroom* category is the largest indoor class, and the smallest are *laundromat* and *staircase*. On the other hand, *parking lot* is the largest outdoor class and *soccer field* is the smallest. Very few images are found in the *construction site*, *soccer field*, *laundromat*, *staircase*, and *storage room* classes due to the limited encounters with these scenes during acquisition trips. The dataset was collected on and around a university campus.

**Figure 4:**
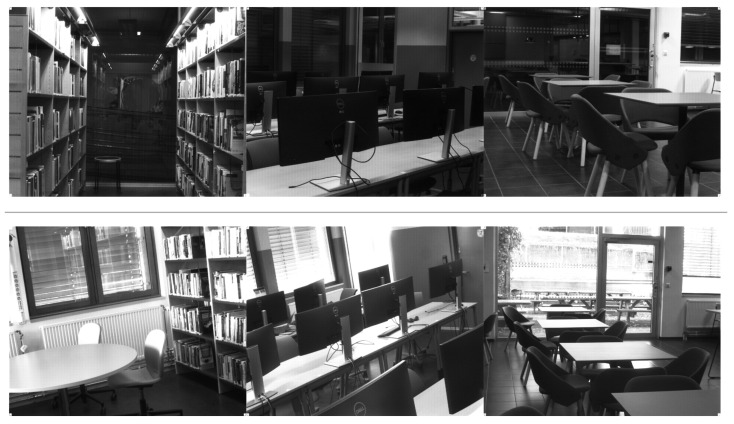
Examples of similar scenes taken during day time and night time. Top row corresponds to images taken at night under artificial lighting and bottom row corresponds to images taken during the day time.

Images of *library*, *office*, and *restaurant* classes were captured under varying lighting conditions. These classes have high dynamic range conditions with daylight entering through the windows, while the camera is exposed to the indoor light level. The dataset also contains images captured at night in artificial lighting. Examples of these images are shown in Figure 4.

The category naming scheme of the Places dataset [21] was followed, with the *bike stand* class being an exception, as it is not present in the Places dataset. This scheme was chosen for its convenience in training Places-CNN with this dataset, given that Places is a widely recognized large-scale scene recognition dataset.

## 3. Methodology

**Figure 5:**
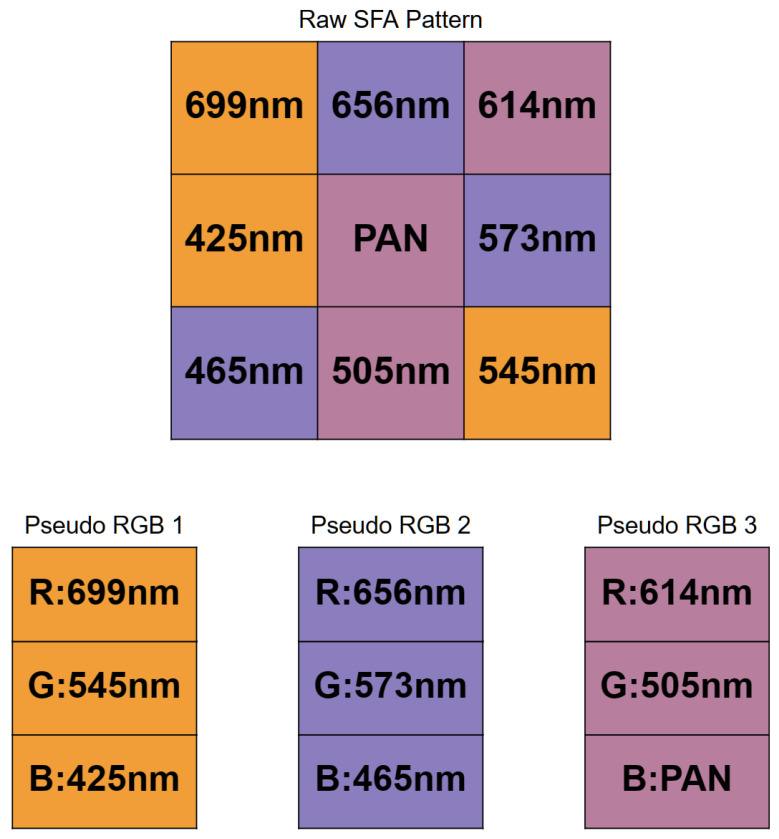
Selected bands that form pseudo-RGB pixel in each pseudo-RGB image.

**Figure 6:**
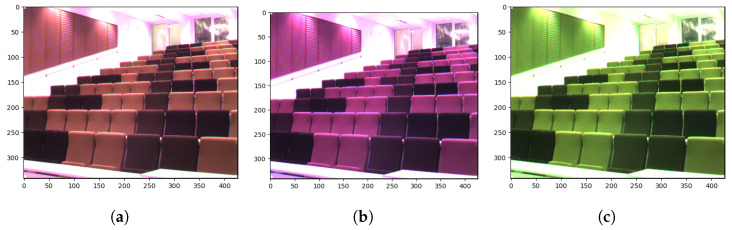
Example Pseudo-RGB images. (**a**): Pseudo-RGB 1. (**b**): Pseudo-RGB 2. (**c**): Pseudo-RGB 3.

This section covers the details of the proposed method for classifying scenes in raw SFA images. The proposed model is based on Places-CNN [20]. The model is not trained on the raw SFA dataset; instead, the pretrained weights of the Places-CNN are used. For details of the Places-CNN training methodology, we refer to [20]. Places-CNN is trained on RGB images of the Places dataset [21] so it cannot be used readily with the raw SFA images, which consist of one channel. We introduce a three-pathway network which accepts three pseudo-RGB images, performs inference on each image independently, and finally combines the Softmax probability scores. The three RGB images are obtained from the 9-band raw SFA image. This scheme enables full utilization of the spectral information. Considering the raw SFA to be a grayscale image reformulates the problem and shifts the multispectral aspect to be implicit in the model. It also makes the model applicable to any nine-channel multispectral camera. We selected three bands from the 3 × 3 filter array to create a 3-channel pixel in the pseudo-RGB image. Figure 5 shows the selected bands that form the pseudo-RGB pixels in each of the three pseudo-RGB images. These bands were selected based on their wavelengths that correspond to the red, green, and blue colors in the visible wavelength range. One exception is that the panchromatic band is assigned to the B channel in the pseudo-RGB 3 image. It was assigned because it was left over after all other bands were selected. Figure 6 shows an example of these three pseudo-RGB images. These pseudo-RGB images have a resolution of 427 × 342, while the original raw SFA is 1280 × 1024.

**Figure 7:**
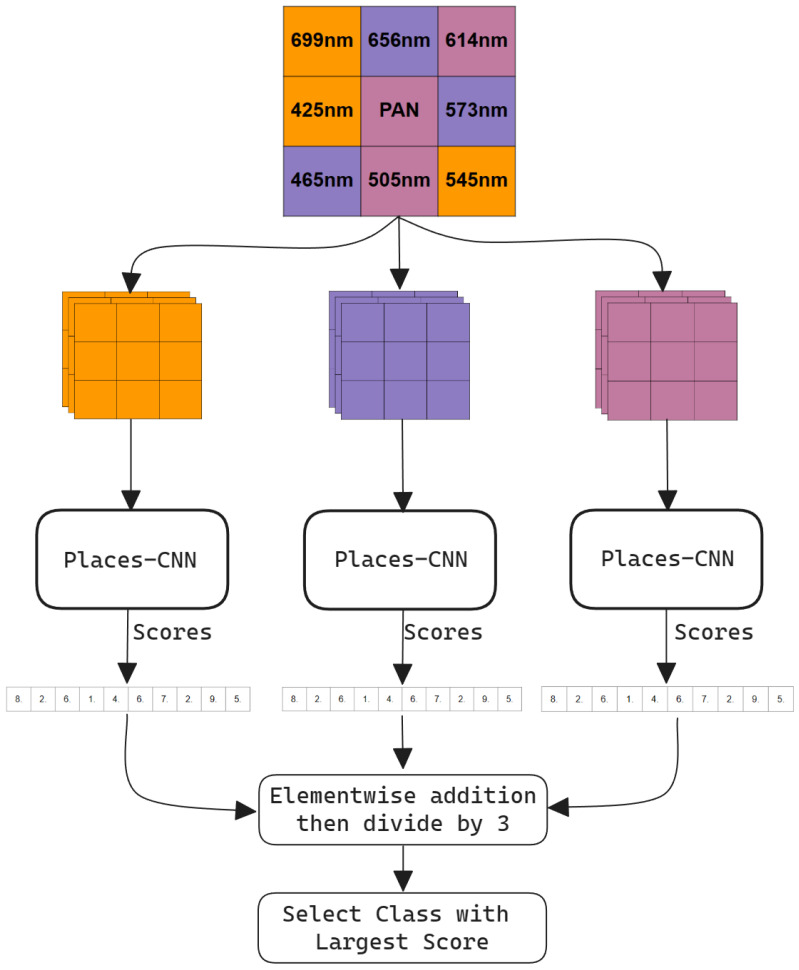
Proposed methodology with Three-Pathway Network.

The proposed model illustrated in Figure 7 takes three input images with three channels, the inference on each image is performed independently by a pretrained 11 million parameter Places-CNN network, and finally the prediction is calculated by combining the softmax probabilities of all three networks and selecting the class with the highest score. The Places-CNN architecture is a residual network with skip connections [33] consisting of 18 residual layers. All three networks have shared weights and return a 365 length vector of Softmax probabilities corresponding to each class of the Places dataset. The three resulting probability vectors are summed element-wise and then divided by three to normalize back to the 0 to 1 range. Then, this normalized vector is sorted in descending order, and the highest scoring class is picked.

**Figure 8:**
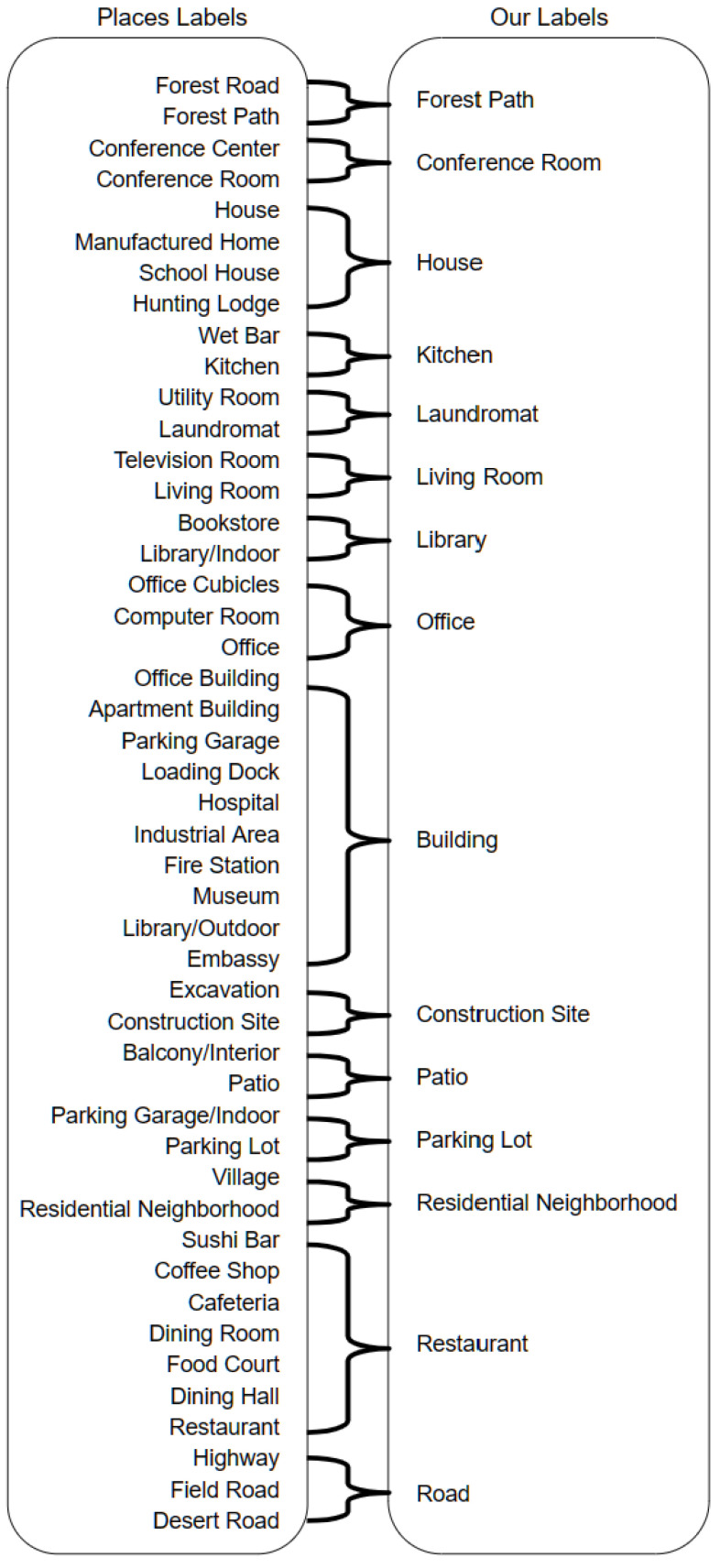
Mapping of Places dataset labels to our raw SFA dataset (CID:Places) labels.

The Places dataset on which Places-CNN is trained contains 365 fine-grained classes. It includes specific classes such as *apartment building*, *office building*, *hospital*, etc. Our dataset has 24 general classes, including those that do not exist in Places (*bike stand*). Therefore, it encapsulates all buildings in the *building* class that does not exist in the Places dataset. To solve this mismatch, a label mapping is performed before combining the scores. So, all specific classes are replaced with general classes that exist inside our dataset, and their scores are summed. All Places dataset labels are analyzed, and the visually and semantically similar classes are mapped to the general class label in our dataset. Figure 8 shows all the label mappings from the Places dataset labels to the labels of our dataset.

Indoor vs. outdoor binary classification is also performed. The Places dataset assigns an additional indoor/outdoor label to the scene class label. After inference, to predict whether the image is of an indoor scene or an outdoor scene, the first 10 largest scores and their corresponding classes are taken and a majority vote of indoor/outdoor labels determines the resulting category.

## 4. Results

In this section, experiments are performed to assess the effectiveness of using pretrained Places-CNN and the proposed three-pathway network for scene recognition in raw SFA images. Accuracy and F1 scores are considered for both indoor vs. outdoor classification and scene classification. Six models with different configurations are compared. Class activation maps returned by Places-CNN are also examined to explain the judgments.

**Figure 9:**
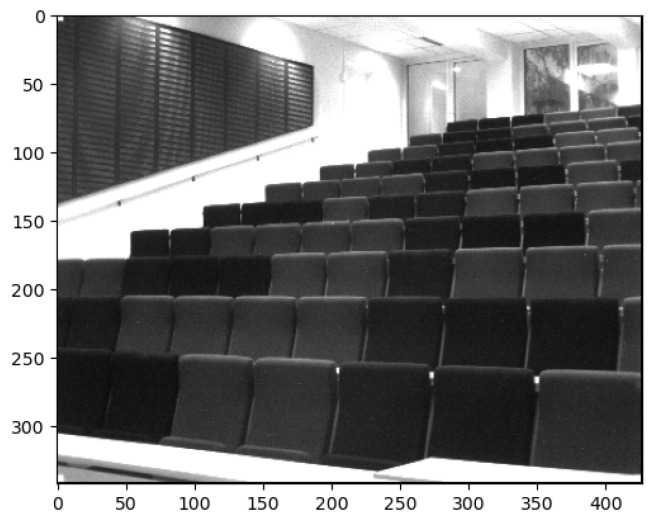
Example grayscale image.

The details of the configurations compared are as follows:

**Config** **1:** **Raw SFA image as input to Places-CNN.** The raw SFA image is a single channel image with the mosaic patterns indicating the 9 bands. It is treated as a grayscale image. The single channel is duplicated along the *z*-axis to obtain a three-channel image. It is sent to the unmodified pretrained Places-CNN for inference.
**Config** **2:** **Pseudo-RGB 1 image as Input to Places-CNN.** The first pseudo-RGB image constructed by selecting band 699 nm as R, 545 nm as G, and 425 nm as B as shown in Figure 5 is sent as input to Places-CNN for inference and metrics are computed.
**Config** **3:** **Pseudo-RGB 2 image as Input to Places-CNN.** The second pseudo-RGB image is used as input to the unmodified Places-CNN.
**Config** **4:** **Pseudo-RGB 3 image as Input to Places-CNN.** The third pseudo-RGB image is used as input.
**Config** **5:** **Grayscale image as Input to Places-CNN.** The middle panchromatic channel is taken and a three-channel grayscale image is produced by duplicating the value three times along the *z*-axis. The size is similar to that of the pseudo-RGB images, and the mosaic pattern seen in Configuration 1 is absent. Figure 9 shows an example grayscale image.
**Config** **6:** **Three Pseudo-RGB images as Input to Three-pathway Network.** The proposed method is as follows: three pseudo-RGBs are constructed and sent to the three inputs of the three-pathway network to perform inference on each image independently, and then the results are combined.

**Figure 10:**
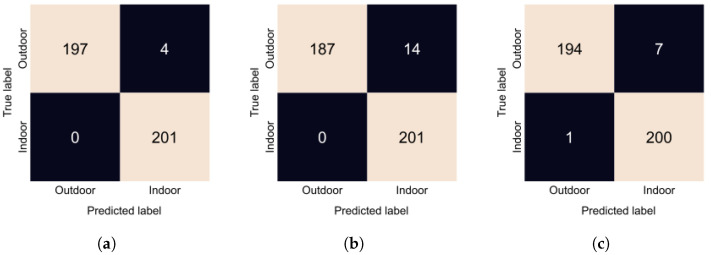
Confusion matrices of the indoor vs outdoor classification task. (**a**): Confusion matrix of Configuration 1. (**b**): Confusion matrix of Configuration 4. (**c**): Confusion matrix of Configuration 6.

Indoor vs. outdoor accuracies and F1 scores are presented in Table 1. All configurations performed very well, achieving almost perfect accuracy. Configuration 1 where we input the raw SFA image performed the best; we can see from Figure 10a that it made only 3 errors. Configuration 4 performed the worst; in Figure 10b, we can see that it incorrectly predicted 14 outdoor scenes as indoor. Finally, Configuration 6, the proposed method, misclassified 5 images as outdoor, as seen in Figure 10c. Overall, the performance of all approaches is very similar.

**Figure 11:**
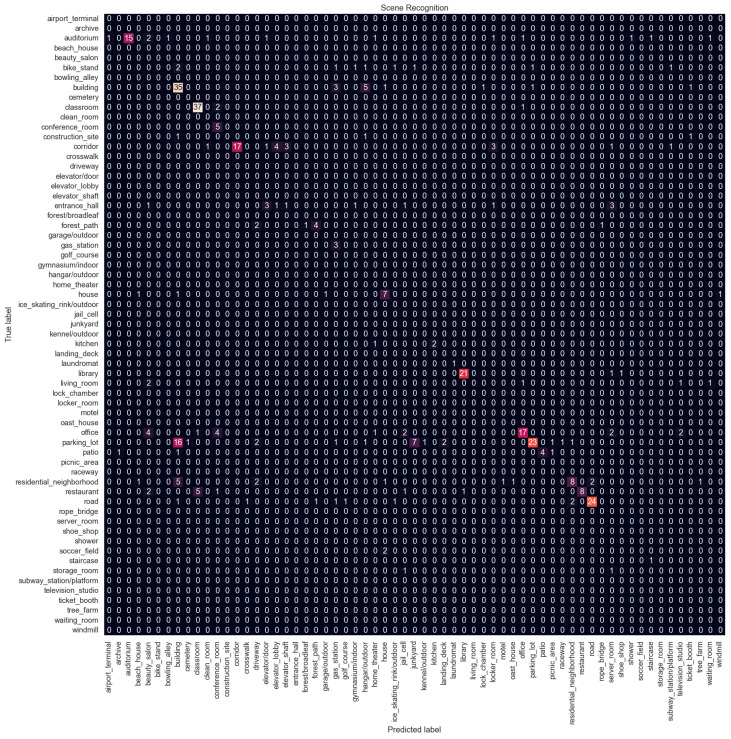
Confusion matrix of Configuration 6 on the scene recognition task.

The performance metrics for the scene recognition task are shown in Table 2. Configuration 1 has the best accuracy, while Configuration 6 has the highest F1 score. Since the dataset for scene recognition is imbalanced, unlike for indoor vs. outdoor classification, the F1 score is the more useful metric here. Figure 11 shows the confusion matrix for Configuration 6 which is the proposed method. Confusion matrices for other configurations are available in Appendix A. Overall, the performance is not good, with the best F1 score of 0.63 and an accuracy of 0.59, and there is a big difference compared to indoor vs. outdoor classification performance.

The model struggles with classes that are related to each other. The parking lot is the most misclassified category. It is confused with the building category. In the dataset, there are many parking lots next to or in front of buildings. The parking lot is also confused with the junkyard. Both categories contain images of cars parked in a line. Similarly, the office is confused with the conference room, the restaurant with the classroom because both have arranged tables and chairs, and the residential neighborhood with the building. The model struggles to distinguish subtle details; for example, the junkyard has cars that are not in good condition, or the restaurants usually have tablecloths and other decorations on the tables while classrooms do not.

Two main reasons for the disparity in performance of both tasks is that the indoor vs. outdoor classification decision is taken with a majority vote of the top 10 scores, while for scene recognition only the top 1 score is considered. Configuration 6 performs better on the more difficult scene recognition task, demonstrating a better bias–variance trade-off based on the F1 score. This is because it combines the decision of three networks. Another reason is that the model is not trained on our dataset, and thus the input is out of distribution for it. Configuration 4 has the worst performance due to the panchromatic channel set to the blue channel, resulting in the most color-incorrect image compared to the other pseudo-RGB images.

**Figure 12:**
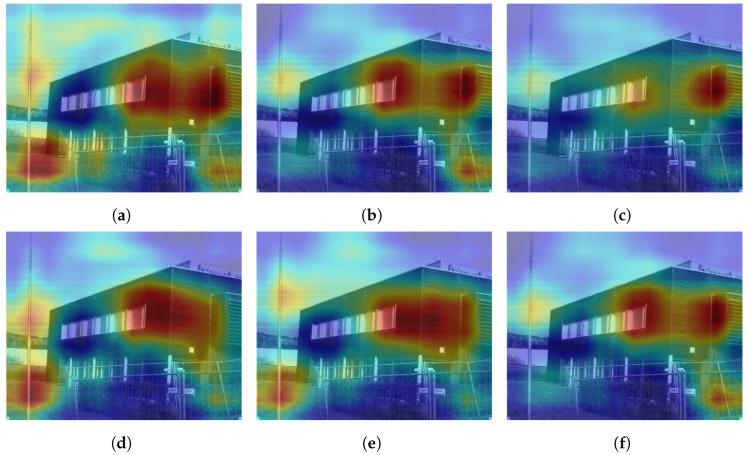
Class activation maps of a *building* image correctly classified by all configurations. (**a**) Configuration 1. (**b**) Configuration 2. (**c**) Configuration 3. (**d**) Configuration 4. (**e**) Configuration 5. (**f**) Configuration 6.

**Figure 13:**
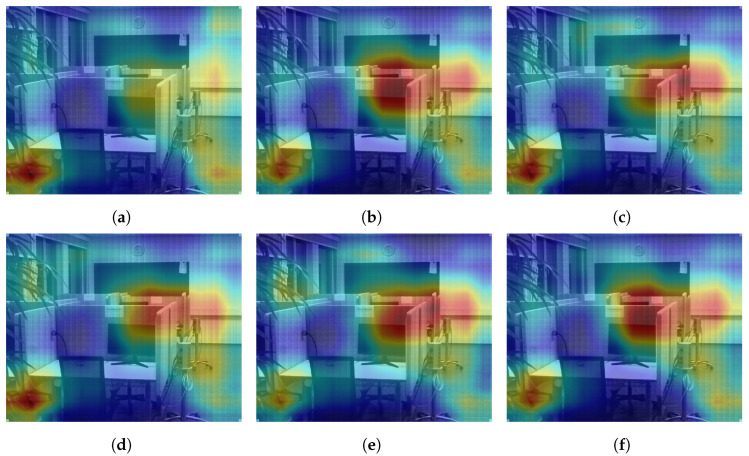
Class activation maps of an *office* image correctly classified by all configurations. (**a**) Configuration 1. (**b**) Configuration 2. (**c**) Configuration 3. (**d**) Configuration 4. (**e**) Configuration 5. (**f**) Configuration 6.

**Figure 14:**
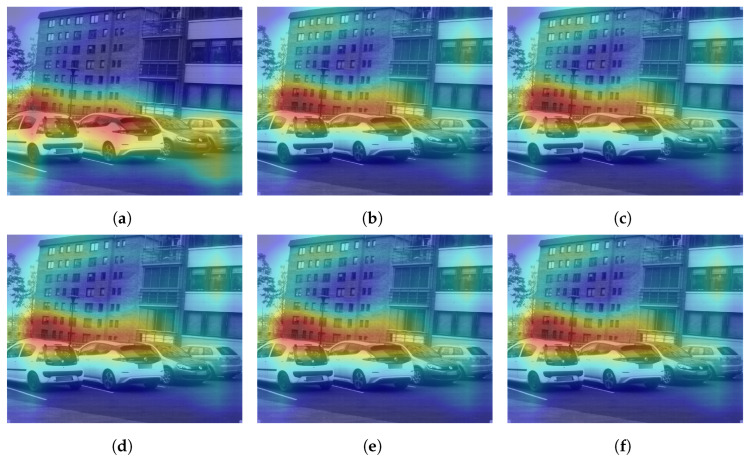
Class activation maps of a *parking lot* image incorrectly classified as *building* by all configurations. (**a**) Configuration 1. (**b**) Configuration 2. (**c**) Configuration 3. (**d**) Configuration 4. (**e**) Configuration 5. (**f**) Configuration 6.

**Figure 15:**
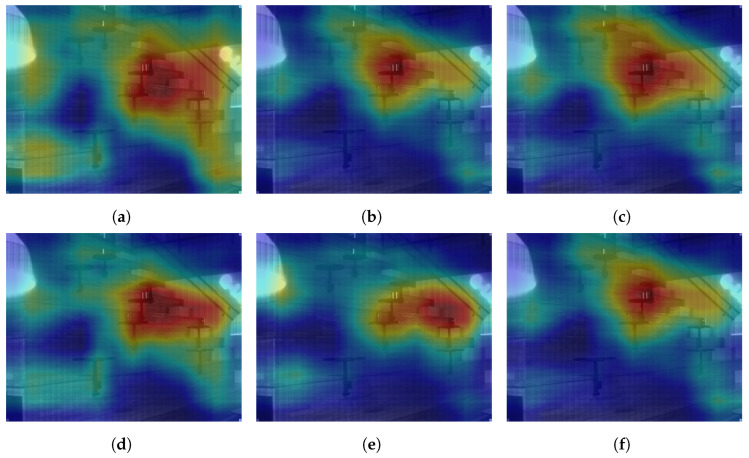
Class activation maps of an *auditorium* image incorrectly classified as *jail cell* by Configurations 1, 4 and 6, as *bowling alley* by Configurations 2 and 3, as *staircase* by Configuration 5. (**a**) Configuration 1. (**b**) Configuration 2. (**c**) Configuration 3. (**d**) Configuration 4. (**e**) Configuration 5. (**f**) Configuration 6.

Further analyzing the configurations, we generate class activation maps from the model. The class activation map is a heat map that indicates which area of the image the model found to be the most discriminating or helpful in its classification. We analyzed class activation maps for two correct classifications and two incorrect classifications, one for the indoor case and one for the outdoor case. Figure 12 shows the class activation maps for an image of a building that was correctly classified by all configurations. All configurations focus on the different parts of the building in the image, which explains their correct predictions. A similar pattern is seen in Figure 13 where the models focus on the display, the cubicles, and the bottom of the revolving chairs to correctly predict the image belonging to the *office* class. Then, we considered misclassification cases. In Figure 14, the image of the parking lot is misclassified as a building. The class activation maps indicate that the models paid attention to the building in the background rather than the cars parked in front. Finally, Figure 15 shows class activation heat maps of an image of an auditorium incorrectly classified. The models focused on the top right of the image, where the staircase and its railing are along with some tables. The misclassifications for this image were varied. Configurations 1, 4 and 6 were classified as *jail cell*, Configurations 2 and 3 as *bowling alley*, and Configuration 5 as *staircase*. The class activation maps did not explain the reason for these predictions. Class activation maps provided some insight into the behavior of neural networks but not the entire explanation. Neural networks remain difficult to explain.

As mentioned earlier, the scene recognition prediction is based on the class with the largest softmax score, while the indoor vs. outdoor classification considers the majority class in the top 10 largest scoring labels. Increasing the top k scores used for the decision improves performance. We considered the example of misclassification shown in Figure 14 where the model predicted the *building* class instead of *parking lot*. The image has the building in the background, while the parking lot is in the foreground. This image can be correctly classified as both *building* and *parking lot*.

**Figure 16:**
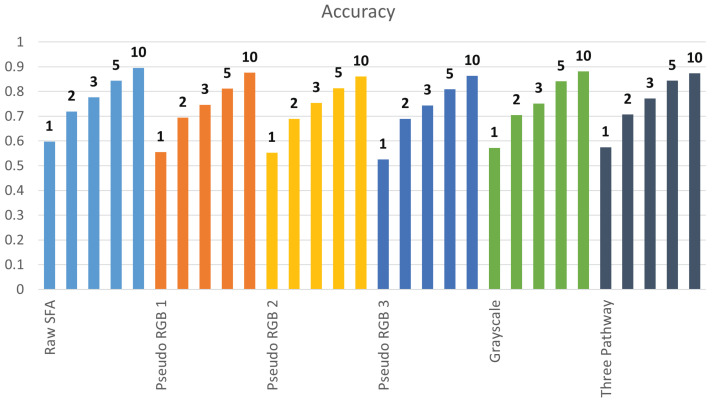

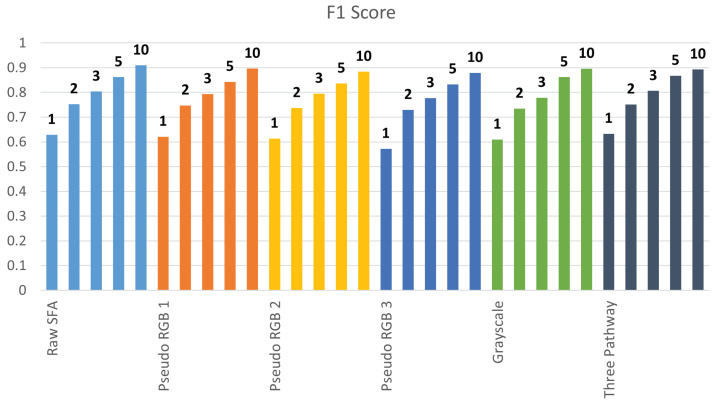
Top K accuracies and F1 scores for each configuration.

Table 3 presents the results in which the top k = 1, 2, 3, 5, and 10 scores were considered and if the correct label was present, the image was marked as correctly classified. As the considered top k scores increase, performance also increases. At the top k = 10, the same level of performance is reached as the indoor vs. outdoor classification. For the top k = 10, Configuration 1 is the best performing configuration, while Configuration 6 is the third best. Figure 16 compares the improvements in accuracy and the F1 score as K increases. There is an improvement of approximately 10% when increasing k by one. The improvement slows to approximately 5% after the top k = 3 and higher. Converting the objective to multi-label classification improves performance. However, it is important to emphasize that this is not needed if the model is trained on the dataset as the highest scoring category is most likely to be the correct one. Reasons for not retraining the model are discussed in Section 5.

For scene recognition, Configuration 1 performs best overall. In Configuration 1, the raw SFA image is duplicated along the z-axis to convert to three channels and input to a pretrained Places-CNN model. The image has a resolution of 1280 × 1024 while the image in all other configurations is smaller at 427 × 342. However, the image in Configuration 1 has mosaic artifacts, whereas the images in other configurations do not. Comparing Configuration 1 and Configuration 5, Configuration 1 still performs better. In Configuration 5, the image is a grayscale image constructed from the panchromatic channel duplicated along the *z*-axis three times. Both images are grayscale (Configuration 1 raw SFA is treated as grayscale), and the difference is in resolution and mosaic artifacts. Table 4 shows the results when the resolution of the grayscale image (Configuration 5) is increased from 427 × 342 to 1280 × 1024 and is compared with Configuration 1. It also shows the result when the resolution of the images in Configuration 1 is decreased to match the images in Configuration 5 (427 × 342). Increasing the resolution of Configuration 5 improves the results slightly, but does not match what is achieved by Configuration 1. Decreasing the resolution of Configuration 1 decreases the results slightly, but not enough, to match the metrics obtained by Configuration 5. More experimentation is required to know why Configuration 1 which has mosaic artifacts works best. Increasing the resolution of images in Configuration 5 improves performance, and decresing the resolution of images in Configuration 1 degrades performance. Resizing images to a bigger size results in blurry images as the process interpolates more pixels. So, if the images in Configuration 5 have a native resolution of 1280 × 1024, they will be sharper and might match the better results of simply using a raw SFA image. The model benefits from the higher resolution of the raw SFA image enough that the noise of mosaic pattern does not cause the performance to be worse than the configurations where the images are smaller.

Further comparisons were made with the selection of other channels to construct a grayscale image. Only one channel was selected from the nine bands and duplicated on the *z*-axis to form a three-channel pixel. The results were similar and can be found in Appendix B.

Configuration 6, which is the proposed model, surpassed Configuration 1 with raw SFA at K = 1, 3, and 5. It was the best performing model at these values of K. The model utilizes the raw SFA image by constructing three pseudo-RGB images and performing inference independently. The results of the three forward passes were combined, and the prediction was chosen. This introduced robustness and reduced noise in the predictions, leading to better results.

## 5. Conclusions

The aim of this work was to assess the effectiveness of using raw spectral filter array imaging for scene recognition. To achieve this, a raw SFA scene dataset was acquired using the SILIOS CMS-C spectral camera and labeled with indoor/outdoor class, as well as scene class following the labels in the Places dataset.

The pretrained Places-CNN was used as the convolution neural network model for scene recognition and indoor vs. outdoor classification. It was trained on the Places dataset with 10 million images and 365 classes. Six configurations (type of input; Configuration 6 also has a different architecture) and variations were evaluated, one of which was a novel architecture that utilized the individual bands of the spectral filter array by separating them into individual images. All models achieved F1 scores above 90% on the indoor vs. outdoor classification task. F1 scores were not good on the multi-class scene recognition task with the proposed model achieving the best score of 63%. Further experiments were carried out to improve performance on the scene recognition task by considering the top K prediction scores for the decision. When K = 10, the scene recognition F1 scores reached 90% for all models.

Experiments were conducted to explain the good performance of Configuration 1. In Configuration 1, the raw SFA image is treated as a grayscale image. The pixels are duplicated along the *z*-axis to form a three-channel image because Places-CNN requires a three-channel image as input. It retains all the spectral information in the image, albeit with redundancy. The raw SFA image contains the mosaic pattern; however, it has the highest resolution of all the other configurations. In Configuration 5, the middle panchromatic channel is selected and duplicated over the *z*-axis to form a grayscale image. These two Configurations are compared because there are visual similarities to explain the affect of presence of mosaic pattern and resolution. It is found that higher resolution leads to better predictions.

Places-CNN was used pretrained on the Places dataset. It was not trained on our custom raw SFA dataset, that is why scene recognition performance was limited when considering only the highest scoring label in the prediction. However, it was not below 50% accuracy, indicating that due to its large-scale training it has the ability to extract relevant features and discriminate them. Places-CNN was not fine-tuned on our dataset because our dataset is highly imbalanced with some classes, such as the laundromat that contains only one image. Fine-tuning on it results in high accuracies and overfitting. The dataset contains 402 images; more images need to be collected to make training a neural network viable.

The pseudo-RGB images were constructed from the selection of the spectral bands from the raw SFA image. More experimentation can be performed to optimize the selection of the bands. Another comparison which was not conducted was with a demosaiced RGB image of the same scenes.

In this work, the role of illuminations was not explored. Further investigation can be carried out to determine whether correcting the illumination in the raw SFA captures has an impact. Higher resolution was found to have a positive impact on performance regardless of mosaic patterns. Further experiments can be conducted to explain this behavior. The Places-CNN model was not trained on the raw SFA dataset. A logical next step is to collect more data and fine-tune the model on it. Additionally, smaller architectures can be explored, such as Mobilenet [34], to make deployment on edge devices possible for real-time applications.

## Figures and Tables

**Table 1:** Accuracy and F1 scores on the indoor vs. outdoor task. The red text indicates the highest values.

Configuration	Accuracy	F1 Score
1: Raw SFA	0.99	0.9901
2: Pseudo-RGB 1	0.9826	0.9829
3: Pseudo-RGB 2	0.9876	0.9877
4: Pseudo-RGB 3	0.9652	0.9663
5: Grayscale	0.9801	0.9804
6: Three-pathway	0.9876	0.9874

**Table 2:** Accuracy and F1 scores on the scene recognition task. The red text indicates the highest values.

Configuration	Accuracy	F1 Score
1: Raw SFA	0.5995	0.6313
2: Pseudo-RGB 1	0.5547	0.6202
3: Pseudo-RGB 2	0.5572	0.6193
4: Pseudo-RGB 3	0.5224	0.569
5: Grayscale	0.5697	0.6064
6: Three-pathway	0.5771	0.6354

**Table 3:** Top K scene recognition accuracy and F1 score. If the label is present in the top k predictions, then the classification is correct. The red text indicates the highest values.

Configuration	Top K	Accuracy	F1 Score
1: Raw SFA	1	0.5995	0.6313
	3	0.7761	0.8043
	5	0.8408	0.8602
	10	0.8955	0.909
2: Pseudo-RGB 1	1	0.5547	0.6202
	3	0.7438	0.7897
	5	0.8109	0.842
	10	0.8731	0.8946
3: Pseudo-RGB 2	1	0.5572	0.6193
	3	0.7562	0.7976
	5	0.8159	0.8391
	10	0.8607	0.8847
4: Pseudo-RGB 3	1	0.5224	0.569
	3	0.7463	0.7785
	5	0.8085	0.8328
	10	0.8582	0.8763
5: Grayscale	1	0.5697	0.6064
	3	0.7463	0.7735
	5	0.8408	0.8614
	10	0.8806	0.8958
6: Three-pathway	1	0.5771	0.6354
	3	0.7711	0.8058
	5	0.8433	0.8674
	10	0.8706	0.8914

**Table 4:** Comparison of scene recognition accuracy and F1 score of Configurations 1, 5, 1 resized to 427 × 342, and 5 resized to 1280 × 1024. The red text indicates the highest values.

Configuration	K	Acccuracy	F1 Score
1: Raw SFA	1	0.5995	0.6313
	3	0.7761	0.8043
	5	0.8408	0.8602
	10	0.8955	0.909
1: Raw SFA (resized to 427 × 342)	1	0.5945	0.6257
	3	0.7711	0.7958
	5	0.8408	0.8602
	10	0.8955	0.909
5: Grayscale	1	0.5697	0.6064
	3	0.7463	0.7735
	5	0.8408	0.8614
	10	0.8806	0.8958
5: Grayscale (resized to 1280 × 1024)	1	0.5697	0.6068
	3	0.7488	0.7763
	5	0.8458	0.8671
	10	0.8781	0.8933

**Figure A1:**
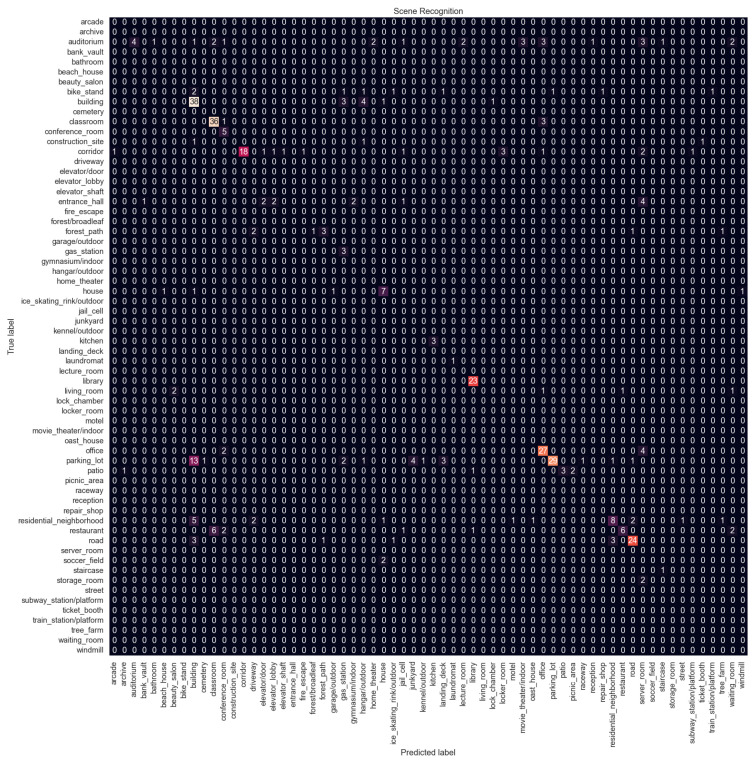
Confusion matrix of Configuration 1 on the scene recognition task.

**Figure A2:**
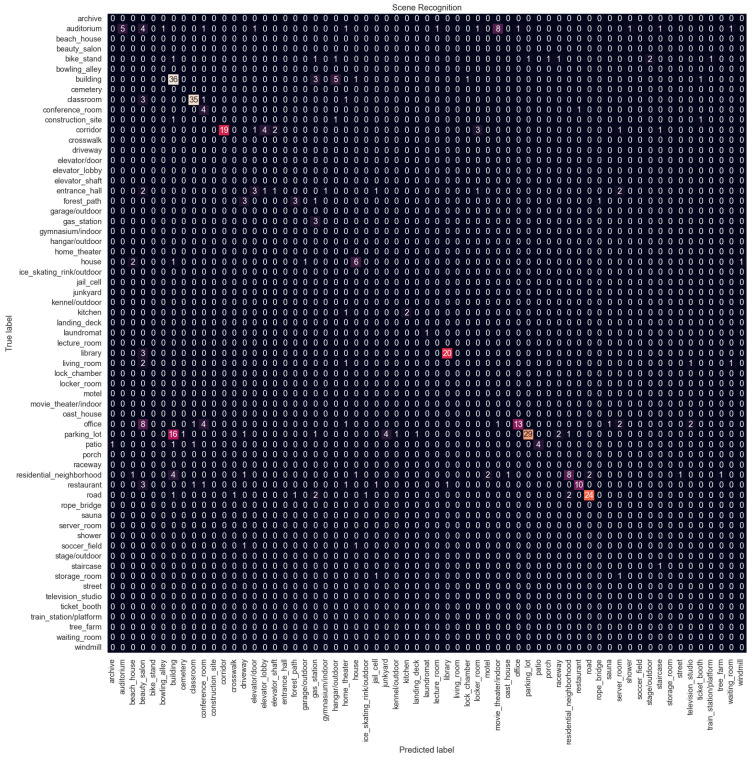
Confusion matrix of Configuration 2 on the scene recognition task.

**Figure A3:**
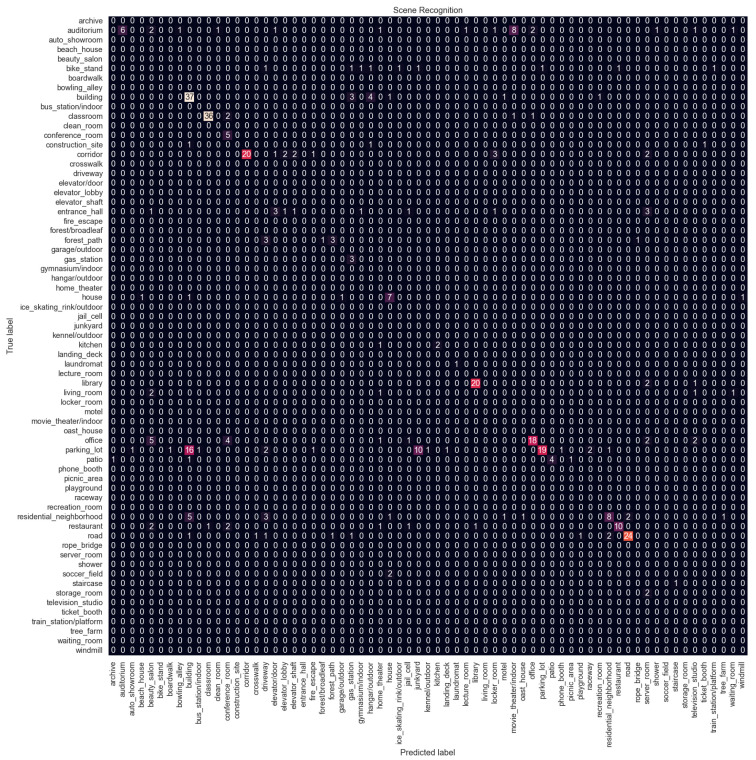
Confusion matrix of Configuration 3 on the scene recognition task.

**Figure A4:**
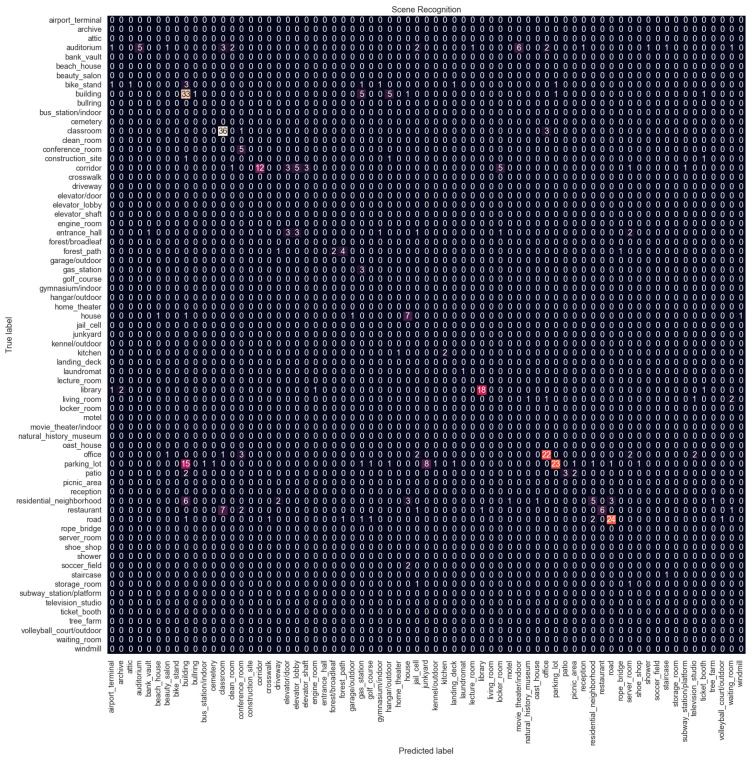
Confusion matrix of Configuration 4 on the scene recognition task.

**Figure A5:**
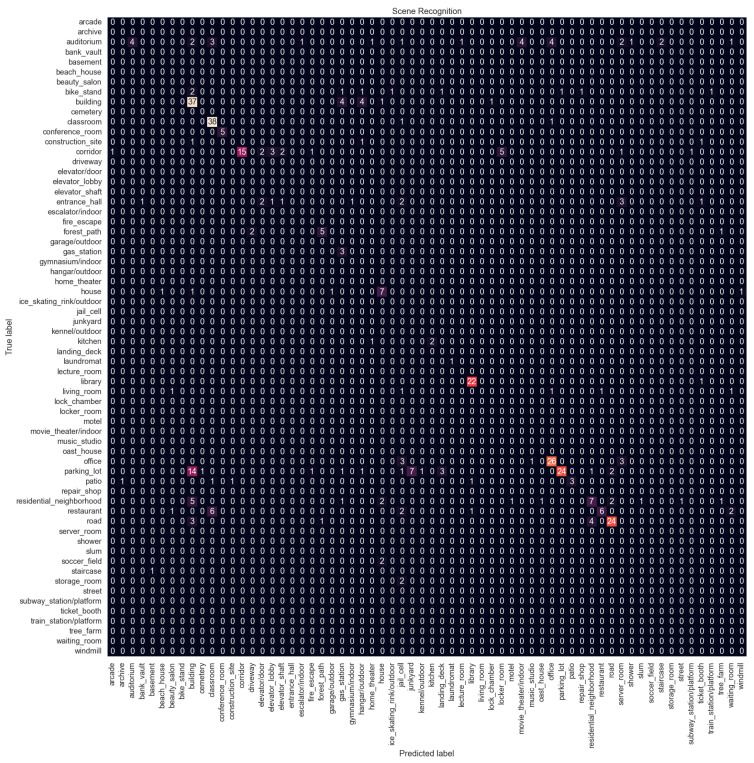
Confusion matrix of Configuration 5 on the scene recognition task.

## Data Availability

The collected raw SFA dataset CID:Places will be made available on the NTNU Colourlab website, http://colourlab.no/cid (accessed on 4 February 2024).

## Abbreviations

The following abbreviations are used in this manuscript:

MDPI	Multidisciplinary Digital Publishing Institute
SFA	Spectral Filter Array
CNN	Convolutional Neural Network
CFA	Color Filter Array
RGB	Red Green Blue
BoVW	Bag of Visual Words
NIR	Near Infrared

## Appendix A. Scene Recognition Confusion Matrices for Other Configs

Scene recognition task confusion matrices for Configurations 1, 2, 3, 4, and 5 are presented in Figure A1, Figure A2, Figure A3, Figure A4 and Figure A5.

## Appendix B. Quantitative Results with Grayscale Constructed by Selecting other Bands

**Table A1:** Comparison of scene recognition accuracy and F1 score for grayscale images constructed by considering different spectral bands. K = 1 for configurations. Red text indicates the best value in the column.

Configuration	Acccuracy	F1 Score
Grayscale (699 nm)	0.5597	0.5896
Grayscale (656 nm)	0.5572	0.5874
Grayscale (614 nm)	0.5572	0.5972
Grayscale (425 nm)	0.5746	0.6069
Grayscale (PAN)	0.5697	0.6064
Grayscale (573 nm)	0.5597	0.6015
Grayscale (465 nm)	0.5696	0.5976
Grayscale (505 nm)	0.5696	0.5989
Grayscale (545 nm)	0.5672	0.6089
